# Effects of the S42 residue of the H1N1 swine influenza virus NS1 protein on interferon responses and virus replication

**DOI:** 10.1186/s12985-018-0971-1

**Published:** 2018-03-27

**Authors:** Jinghua Cheng, Chunling Zhang, Jie Tao, Benqiang Li, Ying Shi, Huili Liu

**Affiliations:** Department of Animal Infectious Disease, Institute of Animal Science & Veterinary Medicine, Shanghai Academy of Agricultural Science, No. 2901 Beidi Road, Minhang District, Shanghai, People’s Republic of China

**Keywords:** Swine influenza virus, NS1 protein, Amino acids substitution, Interferon responses

## Abstract

**Background:**

The influenza A virus non-structural protein 1 (NS1) is a multifunctional protein that plays an important role in virus replication, virulence and inhibition of the host antiviral immune response. In the avian influenza virus or human influenza virus, specific amino acids of NS1 have been shown to be important for the virus to antagonize host antiviral defenses and promote viral replication. However, little research has been reported regarding the swine influenza virus (SIV) NS1 protein.

**Methods:**

To study the effects of the key amino acids of NS1, we rescued NS1 mutants (S42P, D92E, and S42P/D92E) of the A/swine/Shanghai/3/2014(H1N1) strain and compared their replication ability and cytokine production as well as the intracellular localization in cultured cells.

**Results:**

We found that the S42P and D92E mutation displayed no changes on NS1 nuclear localization. The S42P (but not D92E) mutation suppressed protein synthesis and reduced virus growth properties, and there was an inability to antagonize host cell interferon production and IRF3 activation, which led to high levels of IFN-α and IFN-β production.

**Conclusion:**

We conclude that the S42 residue of the NS1 of the A/swine/Shanghai/3/2014(H1N1) strain is the key amino acid in regulating the host IFN response by blocking the activation of IRF3 and thus facilitates virus replication.

## Background

Swine influenza (SI) is an acute respiratory disease caused by the influenza A virus (IAV), which is a member of the *Orthomyxoviridae* family of RNA viruses. The genome of IAV is composed of eight segments of negative-sense single-stranded RNA encoding 10 proteins: hemagglutinin (HA), neuraminidase (NA), M1 matrix protein (M1), M2 ion channel protein (M2), nuclear protein (NP), nonstructural protein (NS1 and NS2), and the RNA polymerase complex (PB1, PB2, and PA) [1, 2]. However, several novel influenza A virus proteins have been identified, such as PB1-F2, PB1-N40, PA-X, PA-N155, and others [3]. The influenza viruses of significance in swine are type A, subtype H1N1, H1N2, or H3N2 viruses and infection from these can result in respiratory diseases, poor growth, weight loss, and immunosuppression. Pigs are often considered as a “mixing vessel” for the generation of reassortant influenza viruses because they have a wide susceptibility for human and avian influenza virus infection [4].

Non-structural protein 1 (NS1) is a virulence factor of the influenza virus and is expressed in the nucleus and cytoplasm of host cells during infection. Functional as a dimer, the NS1 comprises an N-terminal dsRNA-binding domain and a C-terminal protein-binding effector domain [5]. It can interact with a diverse range of cellular factors to antagonize host antiviral defenses and promote viral replication [6]. Mechanisms involved in the NS1 protein’s ability to counteract innate immune responses include the inhibition of cellular pre-mRNA splicing and polyadenylation [7], which blocks posttranscriptional RNA processing and nuclear export, decreases retinoic acid-inducible gene 1 (RIG-I) activation through inhibition of tripartite motif family 25 (TRIM25)-mediated RIG-I ubiquitination, interferes with IFN signaling [8, 9], and directly inhibits specific ISGs such as protein kinase R (PKR) and RNase L [10].

The NS1 proteins of most influenza A virus strains have an average length of 230 amino acids. Point mutation is used to study the key amino acids in NS1 and some of them have been identified to influence the replication efficiency, virulence or host immune responses. Among them, the effect of amino acid residues at position 42 and 92 has been reported in many subtypes of influenza virus. Jiao et al. reported that the amino acid at position 42 of NS1 plays an important role in the ability of H5N1 influenza viruses to antagonize the host IFN response and in the virulence of H5N1 avian influenza virus in a mammalian host [11]. The glutamic acid (E) at position 92 of NS1 in the H5N1 influenza virus was shown to be critical in conferring virulence and resistance to antiviral cytokines in pigs [12]. These conclusions were obtained from avian influenza origin, and there are still no reports on the NS1 protein of swine influenza virus. Therefore, we would like to explore the effect of amino acid residues at position 42 and 92 in the NS1 protein of swine influenza virus.

In this study, we constructed and rescued the NS1 mutant (S42P, D92E, and S42P/D92E) of the A/swine/Shanghai/3/2014(H1N1) strain and explored the effect of the mutant site on the cellular localization and the production of cytokines. Our data indicate that the mutations at these two positions have minimal effects on NS1 nuclear localization; however, the mutant virus with S42P of NS1 reduced growth properties in the cell culture, couldn’t antagonize the host cell interferon production and increased p-IRF3 levels. This work will be useful for a further understanding of how the amino acid mutations influence the function of the NS1 protein and the interaction between H1N1 SIV and the host innate immune responses.

## Methods

### Cells, viruses and antibodies

The A/Swine/Shanghai/3/2014(SH/2014) H1N1 strain was isolated from a clinical pig with the symptoms of swine influenza. Madin-Darby canine kidney (MDCK) cells and the human epithelial kidney cell line (293 T) were used to rescue reassortant viruses from plasmids and were then cultured in Dulbecco’s modified Eagle’s medium (DMEM) (GIBCO, Grand Island, NY, USA) supplemented with 10% fetal bovine serum (Invitrogen, Carlsbad, CA, USA) plus antibiotics. Mouse monoclonal antibodies against IRF3 and phospho-IRF3 (p-IRF3) were purchased from Cell Signaling Technology (Danvers, MA, USA), and a mouse monoclonal anti-actin antibody was purchased from Sigma-Aldrich (St. Louis, MO, USA). Horseradish peroxidase (HRP)-conjugated goat anti-mouse secondary antibody was purchased from Jackson ImmunoResearch (West Grove, PA, USA). Alexa Fluor 488-labeled goat anti-mouse IgG was purchased from Beyotime Biotechnology (Nantong, China). A mouse polyclonal antibody against the NS1 protein was kindly provided by Professor Ying Fang at Kansas State University, USA.

### Generation and propagation of mutant viruses

Wild-type (wt) SH/2014 was created by plasmid-based reverse genetic technology. The cDNAs of the eight gene segments of SH/2014 were cloned into plasmid pBD [13], confirmed by DNA sequencing, and named pBD-PB1, pBD-PB2, pBD-PA, pBD-HA, pBD-NP, pBD-NA, pBD-M, and pBD-NS. Mutations were introduced into the plasmid pBD-NS using overlap-PCR to generate the three mutant NS segments: pBD-NS S42P, pBD-NS D42E, and pBD-NS S42P/D92E. The recombinant virus rSIV, rSIV NS1 S42P, rSIV NS1 D92E and rSIV NS1 S42P/ D92E were generated by co-transfection of eight reverse-genetic plasmids with or without the substitution plasmids pBD-NS into 293 T cells [13, 14]. Eight plasmids (1 μg of each) were mixed and incubated with 15 μl of Lipofectamine 2000 (Invitrogen) at room temperature for 30 min. The Lipofectamine-DNA mixture was transferred to 60% confluent 293 T cells in 35 mm dishes and incubated at 37 °C with 5% CO2 for 4 h. Transfection supernatants were replaced with 2 ml of Opti-MEM medium (Invitrogen) plus 2 μg/ml TPCK-trypsin (Worthington, Lakewood, NJ, USA). At 72 h post-transfection, the supernatants were collected and subsequently passaged in MDCK cells for the virus propagation. Viral stocks were aliquoted and subsequently stored at − 70 °C for the additional experiments. The NS genes of the recombinant viruses were sequenced by the Lasergene software package (DNAstar Inc., Madison, WI) to verify that the generated mutations were as expected. The viral titers were determined using the Reed–Münch method and were expressed as the tissue culture infective dose 50 (TCID_50_) per milliliter.

### Western blotting

Six-well plates of 90% confluent MDCK or 293 T cells were mock-infected or infected with each virus. After 24 h, the cell lysates were harvested and lysed in RIPA lysis buffer (Beyotime). Equal amounts of total proteins were separated on SDS-PAGE gels. The protein bands were transferred onto nitrocellulose filter membranes (Millipore, Billerica, MA) and stained with antibodies against NS1, Actin, IRF3 and p-IRF3 followed by HRP conjugated secondary antibodies. The protein bands were detected using enhanced chemiluminescence detection kits (Thermo Scientific, Inc., Waltham, MA, USA).

### Immunofluorescence assay

MDCK cells were seeded onto cover slips in six-well plates and infected with wt or NS1 mutations. At 6, 12, and 24 h post infection (hpi.), the cells were fixed in 4% paraformaldehyde, permeabilized with 0.5% Triton X-100 for 10 min, incubated in blocking buffer, and then stained with mouse anti-NS1 polyclonal antibodies followed by secondary antibodies conjugated to Alexa Fluor 488. The cell nuclei were stained with 4′,6′-diamidino-2-phenylindole (DAPI) (Beyotime). The samples were mounted on a glass slide and visualized under a florescence microscope (Carl Ziess, Oberkochen, German) using a 400× plan objective.

### Real-time RT-PCR

Real-time RT-PCR was performed to measure the mRNA level of IFN-α, IFN-β and TNF-α after virus infection at an MOI of 1. The human β-actin gene served as an invariant internal control. The primers were designed using Primer3 software and were listed in Table 1. The total RNA from 293 T cells was isolated using TRIzol Reagent (Invitrogen) following the manufacturer’s instructions. The cDNA synthesis was performed using a SuperScript III kit (Invitrogen) and an oligo dT primer (Invitrogen). Five percent of the cDNA product was used as the template for real-time PCR in a final volume of 10 μl containing SYBR Premix Ex Taq II (Takara, Dalian, China). The PCR reactions were performed on a 7500 Real Time PCR System apparatus (ABI, Madison, USA). The amplification conditions consisted of 95 C for 30 s and 40 cycles of 95 °C for 5 s, 60 °C for 30 s, and 95 °C for 15 s. The changes in the amount of target mRNAs are presented as fold changes and were calculated using the comparative CT (CT) method as described [15].

**Table 1:** Primers used in this study

Name	Primer nucleotide sequence (5^,^-3^,^)^a^
NS1-F	TATTCGTCTCAGGGAGCAAAAGCAGGGTG
NS1-R	ATATCGTCTCGTATTAGTAGAAACAAGGGTGTTTT
NS1–42-F	GCCGAGATCAAAAGCCCCTAAAAGGAAGAG
NS1–42-R	TCTTCCTTTTAGGGGCTTTTGATCTCGGCG
NS1–92-F	CTACCTAGCTGAGATGACCCTCGAG
NS1–92-R	CTCGAGGGTCATCTCAGCTAGGTAG
IFN-α-F	CTGTCCTCCATGAGATGATCC
IFN-α-R	CTCATGATTTCTGCTCTGACAACC
IFN-β-F	GCTGGAATGAGACTATTGTTGAGA
IFN-β-R	CAGTTTCGGAGGTAACCTGTAAG
TNF-α-F	CGAGTCTGGGCAGGTCTA
TNF-α-R	GTGGTGGTCTTGTTGCTTAA
β-actin-F	TGGGTCAGAAGGACTCCTATG
β-actin-R	CAGGCAGCTCATAGCTCTTCT

^a^Nucleotides that have been changed are shown in underlined

### Luciferase reporter gene assays

The 293 T cells were cultured in 24-well plates and transfected with 100 ng of the NF-κB luciferase reporter (pNF-κB-TA-luc) (Beyotime). To normalize for the transfection efficiency, 10 ng of the constitutive Renilla luciferase reporter pRL-TK (Promega, Madison, WI, USA) was added to each transfection. After transfection for 24 h, the cells were infected with wt or NS1 mutations at an MOI of 1. Then, 24 h later, the cells were harvested to quantify the luciferase activity using the Dual Luciferase Reporter Assay System (Promega) according to the manufacturer’s protocol.

### Computer modeling of the NS1 protein

The 3D model of NS1 and its mutant proteins were obtained from SWISS-MODEL software (http://swissmodel.expasy.org/), and the structure of H6N6 NS1 (PDB code 4OPH) served as the template. The secondary structures, instability index, aliphatic index, grand average of hydropathicity (GRAVY) and antigenic index of each NS1 protein was predicted and analyzed.

### Statistical analysis

The data were expressed as the means ± standard deviations (SD). The significance was determined with the two-tailed independent Student’s t-test. A *p*-value of < 0.05 was considered statistically significant.

## Results

### Rescue of the recombinant viruses with mutations in NS1

**Fig. 1:**
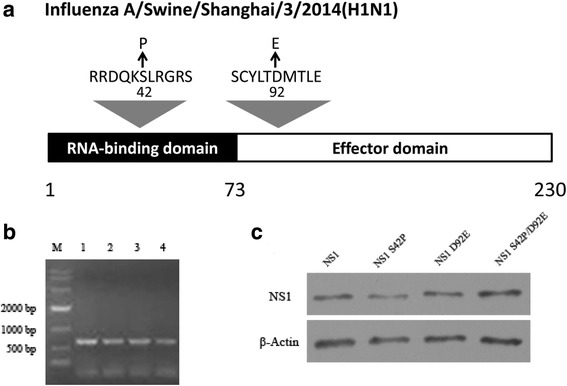
Generation of NS1 mutant viruses. **a** Schematic representation of influenza A/Swine/Shanghai/3/2014 NS1 protein and the mutations employed in this study. **b** NS1 amplification of the WT and NS1 mutant viruses. The viral genomes were amplified using RT-PCR and the length of the NS1 vRNA amplicons was examined using agarose gel electrophoresis. Lane 1, 10,000 bp marker; Lane 2, WT; Lane 3, NS1 S42P; Lane 4, NS1 D92E; and Lane 5, NS1 S42P/D92E. **c** Western blot analyses. Whole cell lysates obtained from MDCK cells infected with mutants (rSIV NS1 S42P, rSIV NS1 D92E and rSIV NS1 S42P/ D92E) and wild-type viruses at an MOI of 0.001 for 24 h were subjected to SDS-PAGE and western blot analysis

To study the effects of the key amino acids on influenza virus replication, the basic amino acids at position 42 serine (S) and 92 aspartic acid (D) of NS1 were mutated to proline (P) and glutamic acid (E) and generated plasmids encoding NS1 S42P and D92E. The rescued recombinant viruses were confirmed by RT-PCR amplification and sequence analysis of the NS1 gene (Fig. 1b). A western blot assay showed that the molecular weight of the NS1 proteins from rSIV NS1 S42P, rSIV NS1 D92E and rSIV NS1 S42P/ D92E viruses were 26 kDa (as expected) (Fig. 1c). These results indicated that rSIV NS1 S42P, rSIV NS1 D92E and rSIV NS1 S42P/ D92E viruses were successfully rescued. Additionally, rSIV NS1 S42P and rSIV NS1 D92E mutations were the result of single nucleotide changes from wild type TCC (S) to CCC (P) and GAC (D) to GAG (E), whereas the rSIV NS1 S42P/ D92E mutation was the result of double nucleotide changes from the wild type.

### Viruses with NS1 S42P D92E mutations made no difference to the NS1 subcellular localization patterns

**Fig. 2:**
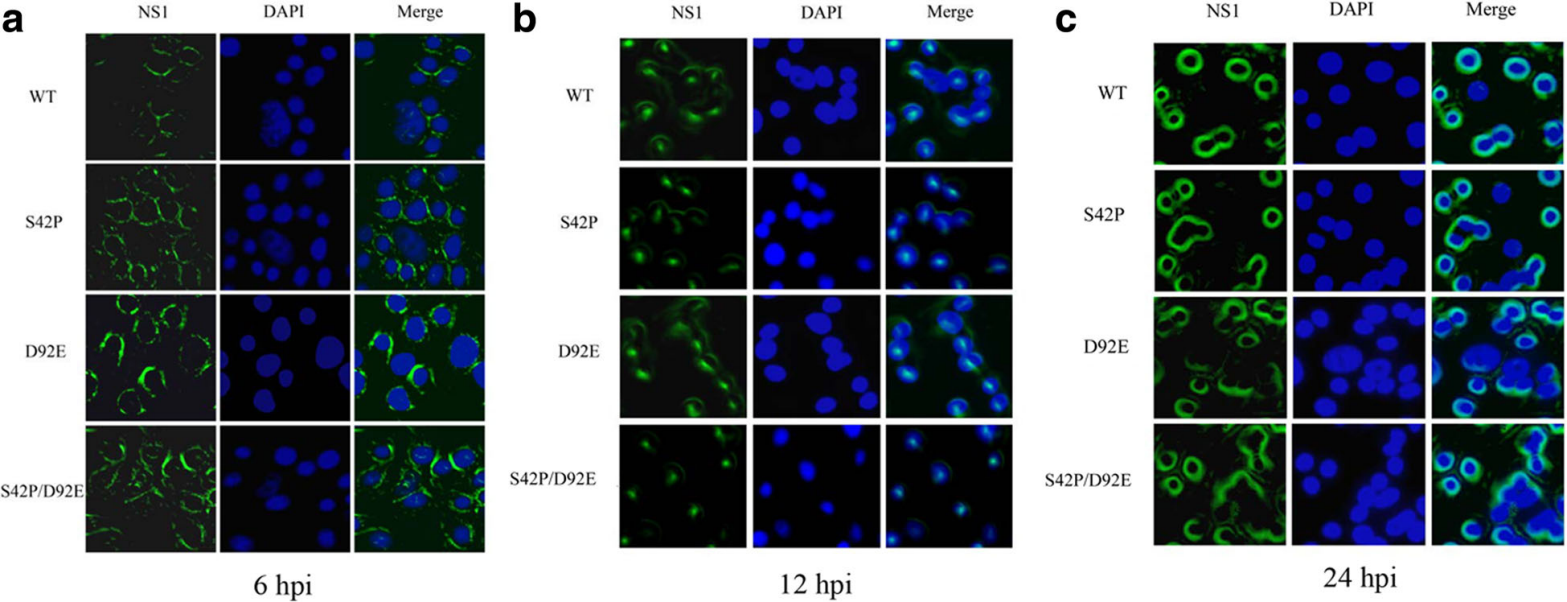
Intracellular localization of NS1 during WT and mutant infection in MDCK cells. MDCK cells were infected with the WT and mutated virus (S42P, D92E, S42P/D92E) at an MOI of 0.001. At 6(**a**), 12(**b**), and 24(**c**) hpi, the cells were fixed and stained with anti-NS1 antibody and DAPI. The localizations of NS1 proteins were visualized using a Nikon Eclipse 80i fluorescence microscope. NS1: stained with FITC-labelled goat Anti-Mouse IgG (green); Nuclei: stained with DAPI (blue); Merge: merged image of double immunofluorescent staining of the same field

To determine whether the P and E substitutions disrupted the NS1 nuclear localization, intracellular localization at the different stages of wt and mutant infection was studied in MDCK cells by an immunofluorescence assay. As shown in Fig. 2, there was no major difference in the localization patterns between the mutated NS1 proteins and the wt protein during the infection. At early stages of infection (6 h), the protein was found mostly in the cytoplasm in all cases (Fig. 2a). At 12 hpi., nucleolar localization was observed in most cells with the infection of both wt and mutated virus (Fig. 2b). At later stages of infection (24 h), most of the mutants and wt virus NS1 proteins again accumulated in the cytoplasm, whereas little was seen in the nucleus (Fig. 2c). Collectively, the NS1 S42P, D92E and S42P/D92E mutant protein behaved very similarly to that of the wt NS1 protein, and thus, these point mutations had no effect on nuclear localization.

### The NS1 mutations S42P attenuated virus replication on MDCK cells

**Fig. 3:**
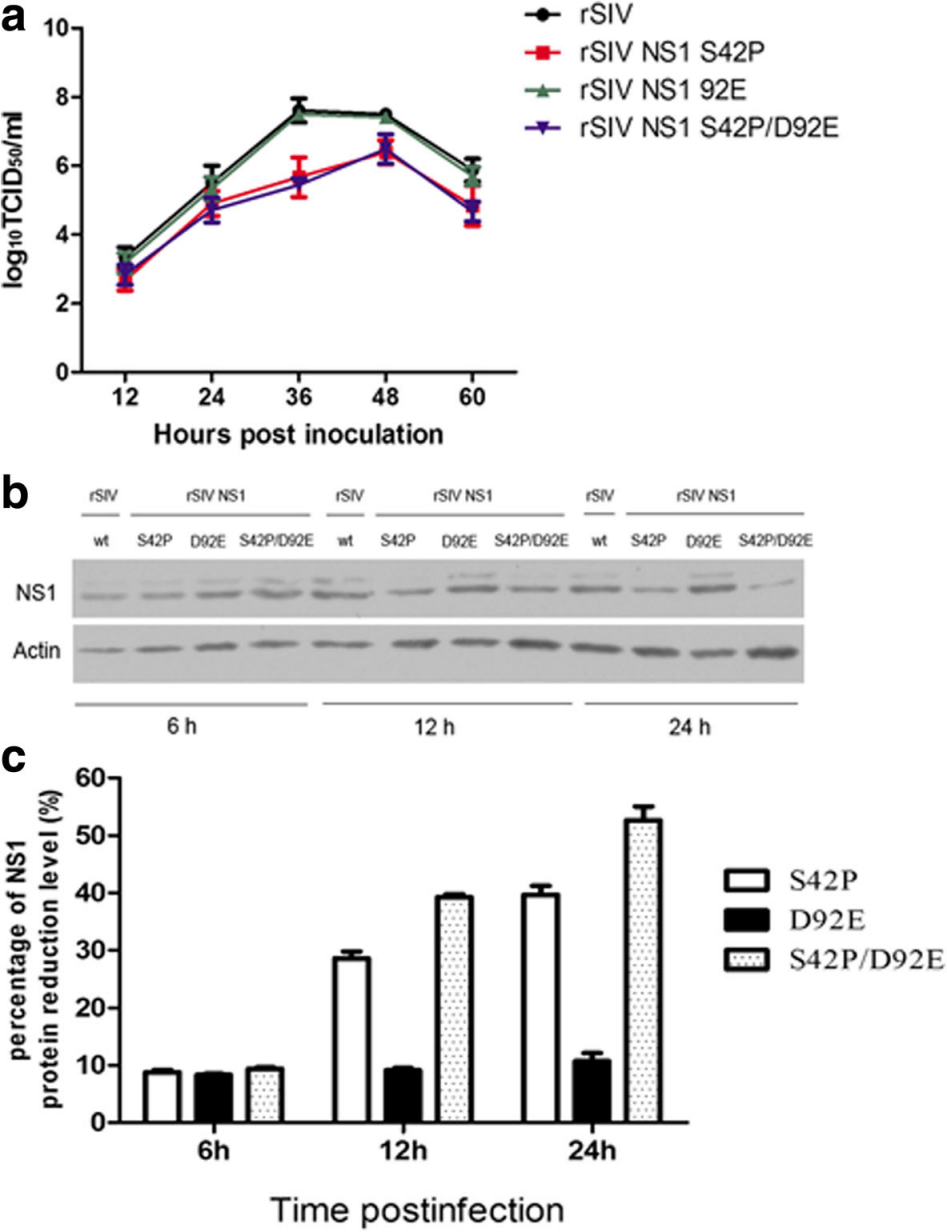
NS1 mutations S42P alter virus replication on MDCK cells. **a** The growth kinetics of the recombinant viruses in MDCK cells. MDCK cells were infected with a wt or mutated virus (rSIV NS1 S42P, rSIV NS1 D92E and rSIV NS1 S42P/ D92E) at an MOI of 0.001. At 12, 24, 48, and 72 hpi, the virus titers in the supernatants were determined using a TCID50 assay. The mean values from three independent experiments are shown for each sample. **b** Expression kinetics of the viral proteins during infection. MDCK cells on 6-well plates were infected at an MOI 0.001 with wt or mutant viruses and the cell lysates that were collected at the indicated time points. The amounts of NS1 and actin in the lysates were examined by western blot. **c** Percentage of the NS1 protein reduction level. The percentage of protein reduction (%) was calculated by the formula [(NS1 expression of wt infected cells - NS1 expression of mutant viruses infected cells)/ NS1 expression of wt infected cells] × 100%; Abscissa: the time points post infection

Next, we analyzed the effect of mutations on the viral growth properties of different recombinant viruses. We infected MDCK cells with the wt and each mutant virus at an MOI of 0.001 and measured the virus titers from samples taken at 12 h intervals. As was shown in Fig. 3a, the titers of rSIV NS1 S42P and rSIV NS1 S42P/ D92E virus were significantly lower than that of wt virus at 36 and 48 hpi., which presented approximately 100-fold lower titers. In addition, the growth kinetics of these two mutants was delayed with a peak titer achieved only at 36 h after infection compared to 24 h with wt viruses. However, the other mutant virus with NS1 D92E displayed almost equal titers with wt virus throughout the infection. To investigate whether the growth defects correlated with any deficiencies in viral protein expression, we infected MDCK cells at an MOI of 0.001 and analyzed the expression of NS1 in cell lysate samples collected at 6, 12 and 24 h post-infection by western blot. As is shown in Fig. 3b, all mutated viruses declined in NS1 expression to a similar extent at 6 hpi, but at 24 hpi, there was a clear reduction of the NS1 level for S42P and S42P/ D92E mutant virus when compared to the wt virus, which revealed a nearly 40% (S42P) and 53% (S42P/ D92E) reduction, respectively, as the band intensities were analyzed with ImageJ software. The percentage of NS1 protein reduction (%) was showed in Fig. 3c. Therefore, mutations of the S42 of H1N1 SIV NS1 protein had been characterized as suppressing protein synthesis and recombinant virus assembly.

### The S42P mutation weakened the effect of NS1 in suppressing IRF3 activation and IFN transcription

**Fig. 4:**
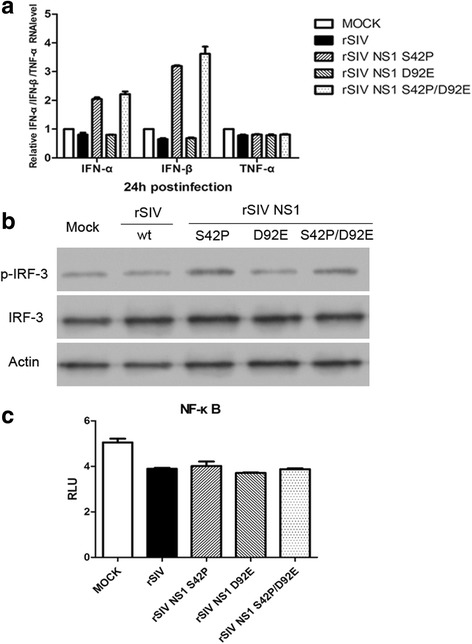
Effect of NS1 mutations on recombinant virus-induced cytokine expression. **a** The 293 T cells were infected with rSIV or NS1 mutant viruses at an MOI of 1 for 24 h. IFN-α/β and TNF-α mRNA levels in the cell lysates were quantified by real-time PCR. The fold changes in the mRNA expression levels were calculated by the comparative CT method. “MOCK” represents samples from uninfected control cells. **b** The 293 T infected with rSIV or NS1 mutant viruses at an MOI of 1 for 24 h were harvested for western blotting analysis of IRF3, p-IRF3 and the β-actin level. **c** The 293 T cells were transfected with pNFκB-luc (100 ng) and then infected with wt or NS1 mutant viruses at an MOI of 1 for 24 h prior to lysis. The constitutive activity of the promoters was measured and is presented as the relative firefly luciferase activity

The potent ability of NS1 to antagonize the IFN system has proven to be an important factor contributing to elevated replication and the virulence of influenza A virus. To analyze whether the reduced replication of rSIV NS1 S42P, D92E and S42P/ D92E mutations is the result of an impaired ability of the NS1 protein to block host cell interferon production, the 293 T cells were infected with the 3 mutant viruses and the wt virus at an MOI of 1, and the production of IFN-α and IFN-β of the supernatants was measured by real-time PCR at 24 h post infection. The results indicated that infection with the rSIV NS1 S42P and S42P/ D92E virus readily induced relatively high levels of IFN-α and IFN-β production compared to the wt virus, which was equivalent to a 2.04- and 3.19-fold increase after rSIV NS1 S42P infection, and a 2.21- and 3.62-fold increase, respectively, after rSIV NS1 S42P/ D92E infection (Fig. 4a). However, infection with rSIV NS1 D92E virus was very weak in its ability to induce IFN-α and IFN-β production into the cell culture supernatant as no obvious difference was observed for the IFN-α and IFN-β mRNA amount compared to wt virus infection.

The NS1 protein of the influenza A virus has been reported to inhibit the activation of IRF-3, which is a key regulator of IFN gene expression. We next examined whether the mutated virus reduced its inhibitory effect of IRF3. To this end, the 293 T cells were infected with either wt or the three mutated viruses, and the cell extracts that were collected at 24 hpi were assayed for the presence of phosphorylated IRF3. The results showed that IRF3 was significantly phosphorylated in cells infected with rSIV NS1 S42P and S42P/ D92E virus, which is consistent with trends of IFN expression (Fig. 4b). These results demonstrated that the amino acid at position 42 was critical for the NS1 protein to inhibit the activation of IRF3 and IFN transcription.

### NF-κB-responsive promoter is not activated by NS1 S42P and D92E virus infection

In addition to type I IFN, innate host responses to viral infection also include secretion of pro-inflammatory cytokines. However, no significant changes in the TNF-α mRNA level were observed in cells between the NS1 mutations (NS1 S42P, D92E and S42P/ D92E) and wt virus at 24 hpi (Fig. 4a). We also assessed the activity of the NF-κB promoter, which has a central position in promoting a variety of pro-inflammatory cytokines. The results indicated that the activity of the NF-κB promoter appeared to be almost at the same level with either NS1 variants or wt virus infection (Fig. 4c). Altogether, these data indicated that the amino acid at position 42 and 92 had little effect on NF-κB-responsive promoter activation.

### 3D models of NS1 mutant proteins are different

**Fig. 5:**
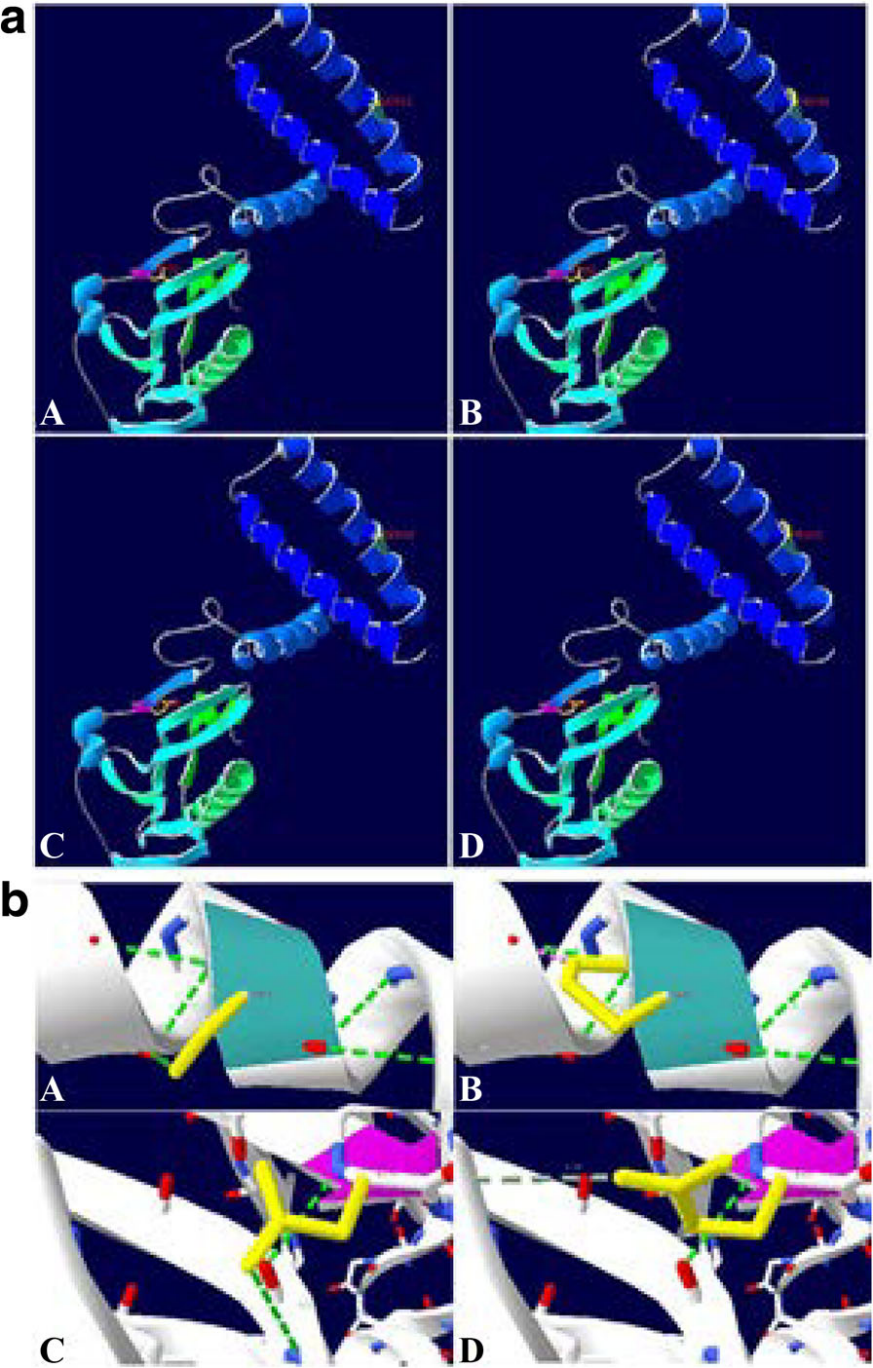
3D prediction of NS1 protein and its mutants. (**a**) The 3D models of NS1 and the mutants predicted by SWISS-MODEL software. A: NS1; B: NS1 S42P; C: NS1 D92E; D: NS1 S42P/D92E. The 42 and 92 amino acid sites were labeled in the protein structural diagrams. (**b**) H-bonds between the mutant amino acid and neighboring amino acids. (A) serine 42; (B) proline 42; (C) aspartic acid 92; (D) glutamic acid 92. The light blue area is the ribbon of the 42 amino acid site, the light pink area is the ribbon of the 92 amino acid site. The green dotted line represents the H-bonds and the numbers represent the length of the H-bonds. The yellow area is the sidechain of 42 and 92 amino acid sites

To observe the protein structure modification after the NS1 mutation, 3D models of NS1 and its mutant proteins were constructed. The mutant of S42P and D92E did not change the structure of the NS1 protein (Fig. 5A). However, the hydrogen bonds of NS1 S42P and NS1 D92E proteins between the 42 (or 92) and neighbor amino acids were changed (Fig. 5B). The grand average of hydropathicity (GRAVY) of NS1 S42P and NS1 S42P/D92E proteins was higher than NS1and the NS1 D92E protein. The instability index of NS1 S42P was the lowest among three mutant proteins. We concluded that the S42P mutant reduced the protein hydropathicity and thus enhanced the stability of the NS1 mutant protein (Table 2). The antigenicity index of amino acid 42 was high (≥1), which indicated it could be a novel antigen epitope (data not shown).

**Table 2:** Characteristic of NS1 and its mutant proteins

Virus isolated	Protein	Instability index (II)	Aliphatic index	(GRAVY)
A/Swine/Shanghai/3/2014 (H1N1)	NS1	65.19	84.83	−0.342
	NS1_S42P_	64.86	84.83	−0.346
	NS1_D92E_	66.03	84.83	−0.342
	NS1_S42P/D92E_	65.70	84.83	−0.346

## Discussion

Influenza A virus (IAV) NS1 is a highly expressed multifunctional protein that plays important roles in viral replication. Mutation or deletion of amino-acid residues at particular sites in the NS1 protein has previously been shown to affect viability, antigenicity, replication efficiency and virulence [13, 16]. Some common variations of NS1 are amino-acid substitutions at particular sites (e.g., S42P, S42G, D92E, D92Y, I106M and A149V) that are responsible for virus growth, the ability to inhibit induction of IFN in vitro and in vivo, and viral pathogenesis in mice [11, 13, 17]. In this study, we rescued a classical swine influenza virus (A/swine/Shanghai/3/2014(H1N1)) and its NS1 protein mutants via reverse genetics and compared their replication ability in cultured cells. The results of the growth curves revealed that at position 42, S-to-P substitution displayed a reduced replication capability in MDCK cells but inhibited viral protein synthesis. Another substitution at position 92 D-to-E had no effect on viral output or viral protein expression. It was possible that the efficient generation of H1N1 swine influenza virus required the presence of Ser at position 42 of the encoded NS1 protein.

Most viruses developed different mechanisms to evade the host antiviral response, and as inhibition of host antiviral defenses is one of the major tasks of the NS1 protein, we questioned whether the attenuated replication by S-to-P substitution might be due to increased induction of antiviral cytokines such as interferons and tumor necrosis factor. We found that substitution of proline for serine at position 42 led to elevated IFN-α and IFN-β transcription levels. The observed effect was attributed to phosphorylation and activation of IRF-3, a related transcription factor that is involved in activation of IFN expression. Although NF-κB is also one of the pivotal regulators of pro-inflammatory gene expression to induce the transcription of pro-inflammatory cytokines [18], we did not find that the activity of the NF-κB promoter and TNF-α mRNA level made a difference with either wt or mutated virus infection. Hence, these findings indicated that an amino acid at position 42 of NS1 was critical for the ability of H1N1 swine influenza virus to inhibit IRF-3 to provide early antiviral responses. In H5N1 influenza viruses, the amino acid at position 42 of NS1 has also proven to be a key amino acid as it plays an important role in preventing the dsRNA-mediated activation of both the NF-κB pathway and the IRF-3 pathway to antagonize the host IFN response [11]. In this study, we first demonstrated that a single amino acid at position 42 of H1N1 swine influenza virus at the NS1 gene played a major role in the resistance to antiviral cytokines. It remains to be determined how the NS1 protein of H1N1 swine influenza virus allows the virus to escape the effects of IFNs.

The presence of glutamic acid at position 92 (E92) in NS1 has been shown to be essential in affecting antiviral cytokine responses in many different subtypes of the influenza virus. According to John, the specific exchange of E for D at position 92 of A/HK/156/97 (H5N1) resulted in an order of magnitude higher quantum yield of IFN [16]. In contrast, Lipatov reported that a reassortant virus harboring a D92E mutation in its H5N1/NS1 gene provoked significantly higher levels of inflammatory cytokines than viruses whose NS1 protein contains D at position 92 [19]. In our study, we used site-directed mutagenesis to substitute aspartic acid (D) for glutamic acid (E) at this position and found that such a substitution had no effect on the ability of the H1N1 swine influenza virus NS1 protein to antagonize the expression of cytokines. We propose that the role of glutamic or aspartic acid at this position may differ from strain to strain.

The RNA/Protein-protein interaction is mainly affected by the hydrophilicity of amino acids inside the protein [20]. The substitution of certain amino acids may change the hydrophilicity of the protein and thus influence protein bioactivity. Since the NS1 S42P mutation showed lower virus titers and higher cellular IFN-β production, we compared the NS1 and NS1 mutant 3D structures, hydrophilicity, antigenic index and instability index. Our structure analysis showed that amino acid mutations at position 42 and 92 changed the hydrogen bonds but had no influence on the structure of the NS1 mutants. Other studies suggest that the single amino acid at S42 residues induces a minor structural change and leads to the loss of function of NS1 [21]. Different structure modeling software and virus strains might contribute to these two controversial results. The hydrophilicity of the NS1 S42P mutant was lower than NS1 [A/Swine/Shanghai/3/2014(H1N1)]. The S42 residue was located within the RNA-binding domain (RBD) and the S42P site mutation decreased its protein hydrophobicity and thus might influence its interaction with RNAs or host proteins. Previous studies found RBD with S42 residue bound double-stranded RNA (dsRNA), whereas that S42P mutation did not [11]. RIG-I acts as a single-stranded RNA sensor and a potential target of viral immune evasion, and the NS1 protein of the influenza A virus blocks the RIG-I activation mediated by viral genomic single-stranded RNA bearing 5′ phosphates [22]. It remains to be investigated what the contribution of the NS1 mutants is to the level of IFN-β associated with the reducing interaction with RIG-I. The D92E substitution increased the instability index and had no effect on the hydrophilicity of the proteins, and D92E was still able to influence the antiviral response by inhibiting IFN-α/β production. Moreover, the antigenicity index of serine 42 of NS1 was over 1, which indicating that it could be an antigen epitope. The amino acid at serine 42 was highly conserved in the human, swine, and equine influenza viruses. Further elucidation of 42 as an epitope would provide new insight into the development of vaccines to control various types of influenza virus.

## Conclusion

In this study, we demonstrate that the S42 residue of the NS1 of the A/swine/Shanghai/3/2014(H1N1) strain is the key amino acid in regulating the host IFN response by blocking the activation of IRF3 and thus facilitates virus replication. This work will be useful for a further understanding of how the amino acid mutations influence the function of the NS1 protein and the interaction between H1N1 SIV and the host innate immune responses.

## Abbreviations

293 T cells: Human epithelial kidney cell line
D: Aspartic acid
E: Glutamic acid
GRAVY: Grand average of hydropathicity
hpi: Hours post infection
MDCK cells: Madin-Darby canine kidney cells
NS1 protein: Non-structural protein 1
P: Proline
p-IRF3: phospho-IRF3
RIG-I: Retinoic acid-inducible gene 1
S: Serine
SIV: Swine influenza virus
wt: Wild-type
